# Biomedical students’ self-efficacy and academic performance by gender in a flipped learning haematology course

**DOI:** 10.1186/s12909-024-05421-2

**Published:** 2024-04-24

**Authors:** Abdulrahman Algarni

**Affiliations:** Department of Medical Laboratory Technology, Faculty of Applied Medical Sciences, Northern Border University, 91431 Arar, Kingdom of Saudi Arabia

**Keywords:** Flipped classroom, Self-efficacy, Academic performance, Gender, Haematology, Medical education

## Abstract

**Introduction:**

This study investigated the impact of flipped learning versus traditional instruction on medical students’ academic performance and self-efficacy in a haematology course, and examined gender differences. Flipped learning is an instructional approach where students review pre-recorded lecture content at home, and active learning occurs in the classroom. Self-efficacy refers to students’ beliefs in their ability to succeed and accomplish learning goals.

**Methods:**

A quasi-experimental study was conducted with 86 third-year Saudi medical students (46 males, 40 females) in a 10-week haematology course. Students were assigned to flipped learning group (*n* = 41) or traditional lecture group (*n* = 45). Both groups completed pre- and post-intervention academic tests and self-efficacy surveys. Data were analyzed using descriptive statistics and t-tests.

**Results:**

The flipped learning group showed an increase in academic scores (*p* <.05) and self-efficacy scores (*p* <.05) compared to the traditional group, but between group differences were not statistically significant. Female students in the flipped learning group showed the greatest increase in academic scores and self-efficacy. Most students perceived flipped learning positively for enhancing learning and preparation for class.

**Conclusion:**

Flipped learning promoted self-efficacy compared to traditional lectures in medical students. Gender-specific benefits were observed, highlighting the need to design instruction to meet diverse student needs.

**Supplementary Information:**

The online version contains supplementary material available at 10.1186/s12909-024-05421-2.

## Background

A considerable amount of the teaching and learning time in medical courses is allocated to didactic or direct teaching methods. Traditional teaching techniques are undeniably basic ways of information transmission. However, according to research in medical education technologies, active learning strategies lead to more active participation by students and larger learning gains than more conventional instructor-centred techniques such as lectures [1]. As a result, medical education has shifted away from traditional, lecture-based teaching and toward approaches that encourage higher-order thinking and active learning. Attempts to maximise the use of the allocated teaching and learning time and cater to the diverse requirements of students by enhancing their active engagement in the teaching and learning process have resulted in various teaching approaches which support active learning techniques, including the evolution of the ‘flipped classroom’ approach [2, 3]. Because of the potential benefits to students’ learning and to teaching practices, the flipped teaching approach has received much attention across a wide range of courses and disciplines [4–6]. Bishop and Verleger [7] explained that this instructional style involves assigning recorded video lectures and instructional activities as homework and then carrying out subsequent in-class activities such as assignments, laboratory work and examinations, reversing the traditional allocation of instructional activities between in-class and assignment settings. During instructional sessions, educators can prioritise the identification and resolution of misunderstandings, foster the cultivation of problem-solving abilities and facilitate student participation. An instructor assumes the role of a tutor or coach, providing guidance and support to students to help them overcome challenges in the application of ideas [8]. The use of flipped classrooms enables educators to spend more time with students, facilitating their progression towards advanced application-based assignments, hence enhancing their learning [9].

Recent research into flipped classroom teaching methods in medical education have demonstrated advantages including improved student preparation [10], active participation, and the promotion of critical thinking skills, as well as disadvantages such as the need for student internet connection and inadequate student preparation [11]. As research on flipped learning in medical education continues to grow, there is a need to evaluate its impacts on student outcomes.

Self-efficacy refers to students’ beliefs in their capabilities to successfully learn and perform academic tasks, which has significant motivational and behavioral influences on learning [12]. However, few studies have examined the effects of flipped classrooms on medical students’ self-efficacy. Furthermore, research on gender differences in flipped learning environments has been limited, despite indications that outcomes may vary between males and females [13].

This study aimed to address these gaps by investigating the impacts of a flipped classroom model on medical students’ academic performance and self-efficacy in a haematology course, and exploring differences based on gender. The research questions examined differences between flipped learning versus traditional lecture groups on test scores and self-efficacy measures, before and after the intervention, for the whole sample and by gender subgroups. Students’ perceptions of flipped learning were also evaluated.

### Literature review

The effectiveness of flipped classroom education on the learning outcomes of university medical students has been contentious in the research conducted to date. The flipped classroom has been applied in various medical subjects such as biology, physiology, haematology and pathology in higher education settings [14]. Empirical investigations in the field of medical education on the use of flipped classrooms, have shown a positive increase in students’ academic performance [15–18], satisfaction [10, 19–21] and their engagement levels [22]. Recent meta-analysis of the effectiveness of the flipped classroom concluded that the approach resulted in a statistically significant improvement in learner performance compared with traditional teaching [23]. In an obstetrics and gynaecology course, for example, Arya et al. [24] found that students in the flipped classroom performed better academically in all medical courses than students in traditional teaching classrooms. A research study in the field of psychobiology found that students who participated in flipped classrooms had enhanced proficiency in collaborative learning practices and showed greater levels of metacognition. Bhatt et al. [18] reported that the findings of pre/post tests administered to internal medicine residents indicated that watching a pre-session video during a medical rounds session led to a statistically significant increase in learning compared with attending a case-based session.

There are grounds to believe that the flipped classroom can boost medical students’ performance. One benefit is that it allows more time for the instructor to clarify concepts and provide constructive feedback, and for students to utilise their acquired knowledge and engage in collaborative efforts with their peers [23, 25, 26]. There is also the fact that having access to recorded lectures enables students to go back over material for more clarification as required, which aids knowledge retention [18, 27]. A meta-analysis in the field of health profession education found that attending flipped classrooms resulted in a significant improvement in student performance compared with traditional education [14, 28].

On the other hand, some studies reported non-significant or neutral outcomes in terms of increasing knowledge [25, 27, 29–31]. For example, a comparative study in the area of anatomy reported that students enrolled in flipped classrooms exhibited comparable performance on assignments involving lower cognitive abilities but demonstrated superior performance on assignments requiring higher cognitive abilities [25]. A recent study by Sourg et al. [31] conducted in the university of Sudan with third-year medical students found that there were no statistically significant differences between the flipped classroom group and and the traditional lecture group in terms of increased medical knowledge. Additionally, an in-depth review conducted in the field of medical education found an absence of strong evidence for the effectiveness of the flipped classroom in boosting knowledge acquisition above and beyond the traditional teaching approach [32]. A similar systematic review of higher education nursing programs found academic outcomes which were either positive or neutral [4].

The impact of the flipped classroom on students’ beliefs in their own abilities (self-efficacy) has been investigated. Self-efficacy is a motivating concept which is both theoretically and empirically well-supported, and it has been shown to have a significant impact on the acquisition of new skills and information. Instructors in the medical field are very concerned about their students’ underlying values and theories of motivation. Self-efficacy among medical students, and its relationship to their growth and development while undergoing their education, is a topic attracting increasing attention [33]. Bandura’s [12] social cognitive theory defined self-efficacy as the belief that one can succeed and the ability to take the steps required to achieve one’s goals, and it plays a significant role in determining achievement outcomes through its dynamic interplay with environmental and behavioural determinants. Students’ confidence in their own talents to put their acquired information and skills to use is often the deciding factor between success and failure in the medical field. Research seems to suggest that one of the most crucial aspects of students’ academic achievement is their sense of self-efficacy. For instance, Chemers et al. [34] stated that students’ self-efficacy in the first year of college is a good indicator of how well they will do in the future. Generally, researchers agree that feeling capable has a beneficial effect on academic performance [33, 35]. Research into students’ perceptions of their self-efficacy has been conducted in flipped medical classes [36–38] and, as expected, the majority of the studies found favourable outcomes. For example, Decloedt et al. [26] observed that flipped classroom students showed elevated levels of self-efficacy in comparison with their counterparts who received conventional instructional methods. Furthermore, Chu et al. [39] found that the level of self-efficacy shown by nursing students was much greater in the flipped learning group than among those receiving the standard teaching strategy. The flipped classroom model seems to be very compatible with enhancing students’ self-efficacy. An example of this is the implementation of the model which necessitates students taking responsibility for their own learning by engaging with course content outside the class [40]. This approach is known to foster students’ sense of ownership over their learning process, a crucial factor in developing self-efficacy [33, 41]. The observed increase reported in the studies identified in the literature could be attributed to the enhanced quality of interactions between students and teachers as well as the increased possibilities for students to witness their peers effectively acquiring skills [12].

In terms of gender differences, researchers have shown that men and women have quite different perspectives about the value of education and, in particular, the value of a science degree [35]. In science education, the results of previous studies examining the correlation between gender and confidence in one’s abilities have been contradictory. Jeong et al. [42] found a range of emotions shown by sophomore students on general science courses in relation to the flipped classroom, with variations seen to be based on gender. The students exhibited notable variations in levels of confidence, with males generally displaying greater levels. On the other hand, the females tended to exhibit higher levels of fear and nervousness than their male counterparts. Yan et al. [43], however, found that female students in the flipped classroom had enhanced preparatory outcomes subsequent to seeing instructional videos before the class. Male students have been shown to have higher self-efficacy than female students in some studies [41, 44] and Gross et al. [13] found gender disparities in examination results in an undergraduate course at the University of Massachusetts, Amherst. The findings showed that female students achieved a more significant improvement in their examination scores as a result of participating in the flipped classroom model compared with their male counterparts. The authors failed, however, to provide any statistical analysis of the findings. A few studies have reported apparent inconsequential gender disparities in science students’ perceptions of their own abilities and confidence in medical courses [45]. Some studies have shown disparities in motivation among students of different genders [46] as well as variations in their view of e-learning [47]. The observed disparities in outcomes between the genders could be attributed to the absence of physical interaction between male and female participants in the online format of the flipped classroom, as posited by Carrick et al. [48], or to the exposure to a wider range of educational resources, as suggested by Gross et al. [13].

Self-efficacy by gender has not been extensively researched in the field of medical education and technology use in a specific model such as the flipped classroom approach. Although there is a wealth of research on technology integration in medical education, specifically interventions on the effectiveness of implementing the flipped classroom in haematology courses [15, 21, 23, 24], few studies [36, 49, 50] have integrated emotional qualities such as self-efficacy by gender into discipline-specific sectors such as haematology. Haematology courses differ from other areas of medical education and are regarded as a core course with a substantial credit allocation in medical education, which underscores their significance in training well-rounded and knowledgeable medical professionals. The purpose of the current study is therefore to evaluate how well the flipped classroom strategy worked to enhance learning outcomes in a haematology course in a Saudi Arabian educational setting. There is a growing recognition of the significance of learners’ affective characteristics in addition to their cognitive attributes, as these factors are considered to be mutually reinforcing and integral to the learning process. Consequently, there is a need for research studies that take these factors into account in order to obtain a comprehensive understanding of learners in medical education programs, specifically in the context of haematology courses.

### Objectives

The purpose of this study is to investigate whether flipped learning has an effect on medical students’ sense of self-efficacy and academic performance and, if it does, whether it varies by gender.

The research questions were:

1. Do haematology test scores differ significantly between the experimental and control groups before and after the flipped classroom intervention?
2. Do self-efficacy scores differ significantly between the experimental and control groups before and after the flipped classroom intervention?
3. Are there significant differences in haematology test scores between female and male students in the experimental and control groups before and after the flipped classroom intervention?
4. Are there significant differences in self-efficacy scores between female and male students in the experimental and control groups before and after the flipped classroom intervention?
5. How do the students who took part in this study feel about the experience of the flipped classroom approach to learning haematology?

## Methodology

### Participants

This study was conducted with medical students at the medical school at the Northern Border University in Saudi Arabia. There were 86 participants: 46 (53.5%) were male and 40 (46.5%) were female. It is important to note that in Saudi Arabia, males and females are educated separately, and there were no mixed-gender classes in the study. The experimental group had 41 students (22 males and 19 females) and the control group had 45 students (24 males and 21 females). Criteria for the selection of participants were based on the convenience sampling technique since the author is a formal assistant professor and the instructor of the haematology course at the Northern Border University. The sample consisted of third year medical students who were enrolled in the haematology course for about 10 weeks during the academic year 2022–2023.

### Data collection process

The design of this study was quantitative quasi-experimental. This interventional study assessed the efficacy of a flipped learning activity which was carried out among medical students. The study took place in the Department of Medical Laboratory Technology over 10 weeks in January 2023. A schedule of the study and haematology content covered presented on appendix A. Students were randomly assigned to either a flipped instruction group (intervention group) or a traditional instruction group (control group). After obtaining informed consent from each student, the participants were accepted into the study. The topics for this study were chosen from the haematology curriculum. Before the experiment, all of the participants took a pre-test designed by the first author (the instructor for this course), with the goal of ensuring that the participants were equivalent in terms of their prior knowledge of haematology before the intervention. A post-test was carried out at the end of the intervention to measure the students’ performance at the end of the course. In addition, a self-efficacy survey was given to all participants to assess their self-efficacy before and after the flipped learning was implemented. Both the pre and the post tests on haematology, as well as the pre and post self-efficacy surveys, were administered to both the flipped group and the traditional group. At the end of the intervention, the students in the flipped group completed a survey to elicit their perceptions on learning haematology with the flipped teaching technique (see Table 1).

**Table 1:** Overview of the data collection process in both groups

Course design	Intervention group	Control group
Pre-training	Two hours training and trial sessions for each flipped group	None
Haematology tests	Pre and post	Pre and post
Self-efficacy survey	Pre and post	Pre and post
Course duration	Ten weeks	Ten weeks
Learning methods	Flipped learning approach	Traditional teaching approach
Activities for each group	Students engaged in both at-home and in-class activities, as outlined below: In the at-home learning environment, students engaged in the preparatory phase by accessing educational content through the use of pre-recorded lectures and online quizzes available on the Blackboard platform. During the scheduled class time, the class started with a student-led discourse on essential concepts, followed by the teacher’s introduction of more intricate inquiries and discussions. The instructor also provided feedback and facilitated collaborative learning activities.	Students were first introduced to the subject matter of a particular week through a series of in-person lectures delivered by the instructor. After receiving instruction during class, students were assigned weekly tasks to do at home.
Instructor’s role	To facilitate, evaluate and synthesise new knowledge	To facilitate and provide knowledge
Assessment strategy	Final exam	Final exam
Students’ perception	Yes	No

### Learning activities in both groups

The flipped groups (male and female students separately) studied using the flipped learning approach. The implementation of the flipped learning was based on Merrill’s ‘First Principles of Instruction’ design theory [51]. The four core principles of task-based learning highlighted by Merrill were that existing knowledge is ***activated*** as a foundation for new knowledge, new knowledge is ***demonstrated*** to the learner, new knowledge is ***applied*** by the learner, and new knowledge is ultimately ***incorporated*** into the learner’s world [51]. Full descriptions of our version of the flipped learning and how each principle was implemented are in Appendix B.

The flipped instruction was divided into two parts. First, online out-of-class learning materials (video lectures, online quizzes and learning resources) were provided through the Blackboard learning platform. There were 10 YouTube videos posted each week on key haematology concepts covered during the study. Each video was followed by a short quiz with three multiple choice questions on the materials presented, with immediate feedback on the quiz answers.

Second, the in-class activities were designed to promote active learning and group-based problem solving without the need to spend time lecturing because the students had already gone through the online materials and were therefore ready for in-class active participation. The instructor was in the class with some of the materials, not to present them but to engage students in greater collaboration and classroom discussions with various hands-on active learning techniques. The students were divided into groups to enhance group dynamics and handouts were provided which contained a series of problem-solving inquiries and case scenarios. Groups were allocated 20 min to engage in conversation. Following this, a debate was conducted using clinical scenarios to foster discussion among the students. The solutions to the scenarios were deliberated upon by the whole class.

The control groups (male and female students separately) learned by following the traditional teaching methods in which instructional time was mostly used to deliver lectures through PowerPoint presentations, and the remaining time was used to facilitate student engagement in classroom activities and address inquiries raised by the students. The instructional content was not made available in an online format and was not distributed before the start of the class.

### Measurements

#### Haematology tests

In the pre- and post-intervention haematology tests, 30 multiple-choice items were administered to the students before and after the flipped learning intervention. Topics covered were components of blood cells (8 items), identifying all organs involved in blood cell production (7 items), integrating various blood cell types with their functions (6 items) and laboratory procedures associated with blood evaluation (9 items) (see Appendix C, D).

#### Self-efficacy scale

The self-efficacy survey contained 23 statements to determine students’ self-efficacy emphasising haematology skills, adopted from Baldwin et al. [52]. The Biology Self-Efficacy Scale was based on the social cognitive theory put forward by Bandura [12]. It is a valid and reliable tool for studying non-biology majors’ confidence in mastering specific course skills [52]. The items encompassed various domains, including the development of skills in reading, summarising and critiquing articles and presentations related to the field of haematology. Additionally, the items involved the ability to effectively explain haematology topics and provide tutoring to fellow students in relevant haematology subjects or courses. The items emphasised the cultivation of a scientific approach to writing and thinking, which encompassed understanding the necessary steps and extrapolating information learned in class to other aspects of one’s life. Each question was scored on a 5-point scale, with 1 indicating ‘not at all confident’, 2 ‘only a little confident’, 3 ‘fairly confident’, 4 ‘very confident’ and 5 ‘totally confident’. The higher the score, the more confident the student is in executing each behaviour. The scale was modified based on the course content. For each question, the students were asked to think about how confident they would be in carrying out a given task (see Appendix E). To further ensure the validity of the survey, the researcher conducted a thorough reassessment of the translated scale in order to determine its suitability in the specific context of Saudi Arabia for the purpose of the current study. The survey was then retested and demonstrated internal consistency, as measured by Cronbach’s alpha, were calculated to be 0.86 for the pre-test and 0.92 for the post-test.

#### Students’ perceptions

Students in the flipped group were given a survey on their perception of flipped classroom instruction with 10 questions using a 5-point Likert scale (strongly disagree, disagree, neutral, agree, strongly agree). The survey was adapted from a validated questionnaire developed by Pierce and Fox [53]. The survey investigated two domains of student perceptions: students’ perceptions of the online class activities via Blackboard and the in-class activities during face-to-face lectures. This survey was modified and revalidated appropriately to suit the context of the study and the needs. The survey was sent to each student in the flipped group through their online university account in Blackboard and was available for 5 days after the administration of the post-haematology test and self-efficacy survey. The survey items measuring agreement (strongly agree and agree) and disagreement (strongly disagree and disagree) were aggregated for the purpose of reporting. Reliability and validity tests proved that this survey was highly reliable and credible. The reliability coefficient for the whole survey was α = 0.89. Only students in the flipped group completed the survey. Descriptive statistics of the students’ perceptions are presented in Appendix F.

#### Ethical considerations

The appropriate ethical approval was obtained from Ethics Committee of the Northern Border University. The research proposal and objectives were explained to all of the participants who had given their voluntary consent. They were told that they had the right to withdraw at any time without any need to explain their decision and they were made aware that they had the rights to benefit, to suffer no harm, to privacy and to confidentiality. Informed consent was obtained from participating students using forms which were sent through Google Sheets to all of the participating students.

#### Data analysis

The acquired data were coded and entered into a Microsoft Excel spreadsheet and analysed using R. Two outcome variables were measured: haematology test scores (‘knowledge’) and self-efficacy test scores. Each participant’s pre-intervention knowledge score was subtracted from their post-intervention knowledge score to generate a knowledge-increase score for each participant. Each participant’s pre-intervention self-efficacy rating was subtracted from their post-intervention efficacy rating to generate an efficacy-increase score for each participant. These two increases in scores were used as the dependent variables. The mean of the knowledge-increase and self-efficacy-increase scores were tested in a one-sample *t* test, with the null hypothesis being that the mean increase of both knowledge and self-efficacy would be zero. This is equivalent to a paired-sample *t* test of the difference between pre-intervention knowledge and post-intervention knowledge. The knowledge-increase scores were also used as the dependent variable in an independent-samples *t* test of the difference between the mean of the intervention group and the mean of the control group. To test whether the effect of the flipped learning intervention on the increase in knowledge and self-efficacy varied by gender, a linear model with an interactive term was used. The dependent variables were the knowledge-increase and the self-efficacy-increase scores. The model included the following predictor variables: group (1 for the intervention group or 0 for the control group), gender (1 for male or 0 for female), and a multiplicative interaction of those two variables.

## Results

**Fig. 1:**
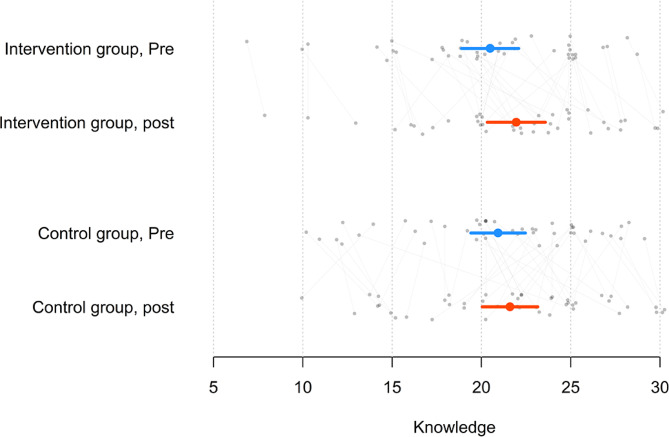
Increase in knowledge before and after the intervention for both groups Note: The circle is the mean, and the line is the 95% confidence interval OR quartiles, and grey dots are individual students

The level of participation in the online activities was high, with students on average accessing 85% of the video lectures and successfully completing 93% of the quizzes. Among the full sample (intervention and control groups combined), knowledge increased by an average of 1.05 points out of a maximum possible score of 30 (*t*_85_ = 2.95, *p* <.05). The mean increase in knowledge was 1.46 among the intervention group (*t*_40_ = 3.08, *p* <.05) and 0.67 among the control group (*t*_44_ = 1.28, *p* =.21). The increase in knowledge was significant in the intervention group but not in the control group. No significant difference was found between the knowledge increase of the intervention group and the knowledge increase of the control group (*t*_84_ = 1.12, *p* =.26) (see Fig. 1).

**Fig. 2:**
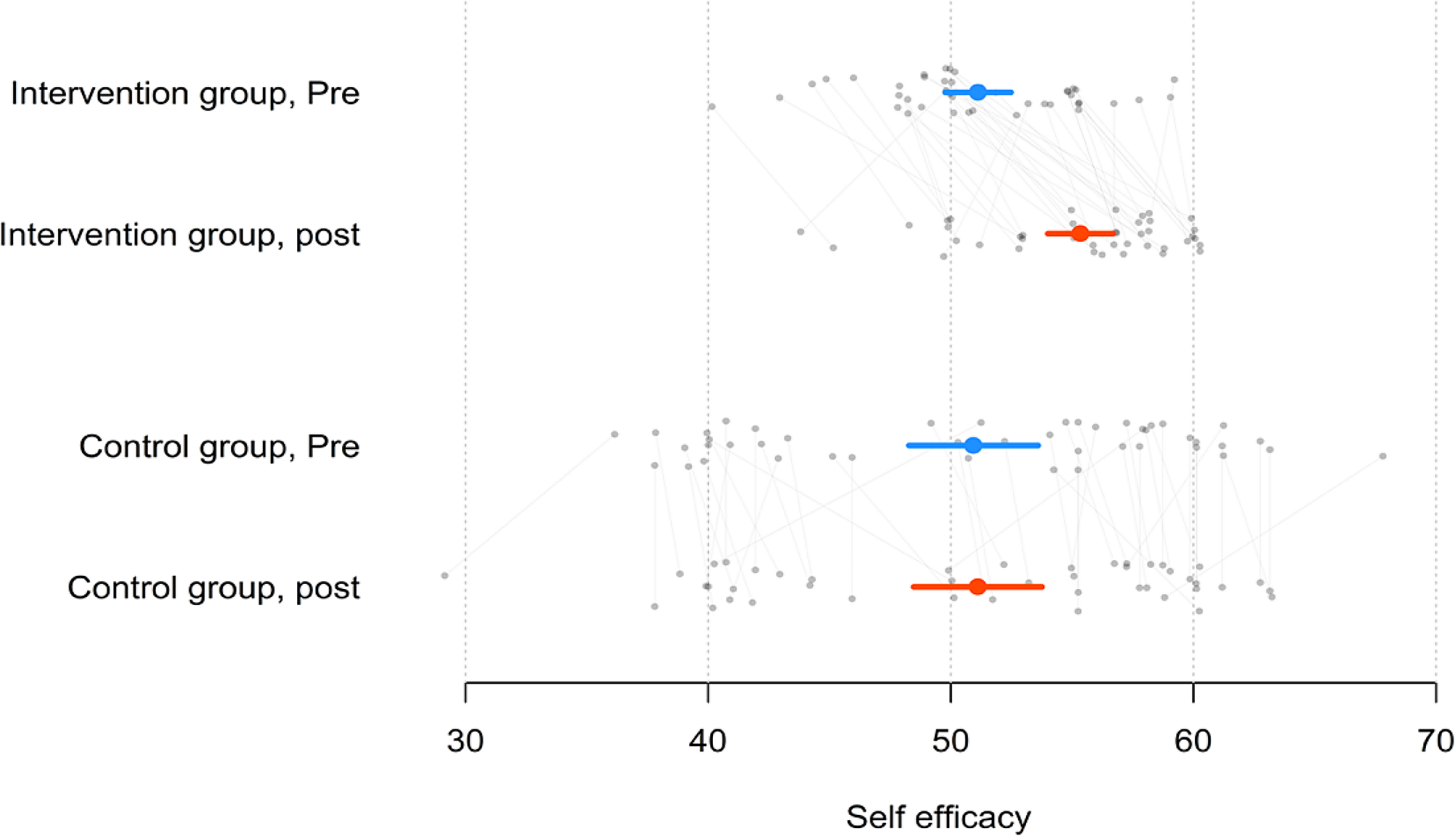
Self-efficacy scores before and after the intervention for both groups Note: The circle is the mean, and the line is the 95% confidence interval OR quartiles, and grey dots are individual students

Among the full sample, self-efficacy increased by an average of 2.10 points (*t*_85_ = 2.79, *p* <.05). The mean increase in self-efficacy was 4.22 among the intervention group (*t*_40_ = 7.59, *p* <.05) and 0.18 among the control group (*t*_44_ = 0.34, *p* =.74). The increase in self-efficacy was significant in the intervention group but not in the control group. An independent-samples *t* test of the difference between the mean increase in self-efficacy of the intervention group and the mean increase in self-efficacy of the control group found a significant difference (*t*_84_ = 5.27, *p* <.05). This implies a significant improvement in the perceived self-efficacy of the flipped group after the flipped learning intervention (see Fig. 2). For the full descriptive statistics, see Table 2.

**Table 2:** Summary of Means and Standard Deviations on outcome measures

Group	Variable– Outcome measures	*N*	Mean	SD
**Intervention**				
	Academic performance pre-score	41	20.49	5.07
	Academic performance post-score	41	21.95	5.16
	Self-efficacy pre-score	41	51.12	4.27
	Self-efficacy post-score	41	55.34	4.24
**Control**				
	Academic performance pre-score	45	20.93	5.09
	Academic performance post-score	45	21.60	5.19
	Self-efficacy pre-score	45	50.93	8.86
	Self-efficacy post-score	45	51.11	8.77

### Gender differences

**Fig. 3:**
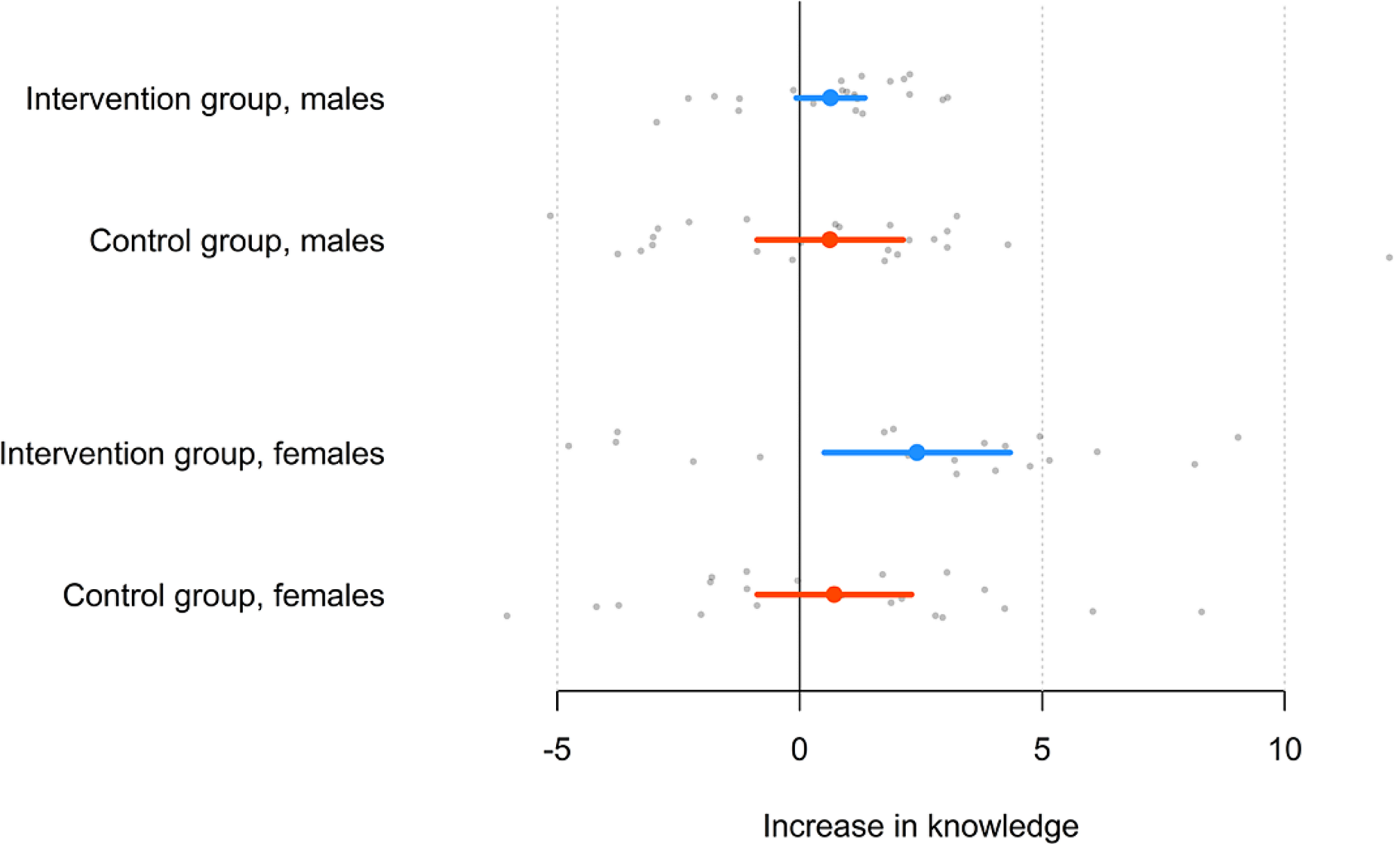
Increase in knowledge for the intervention and control groups for male and female students Note: The circle is the mean, and the line is the 95% confidence interval OR quartiles, and grey dots are individual students

**Fig. 4:**
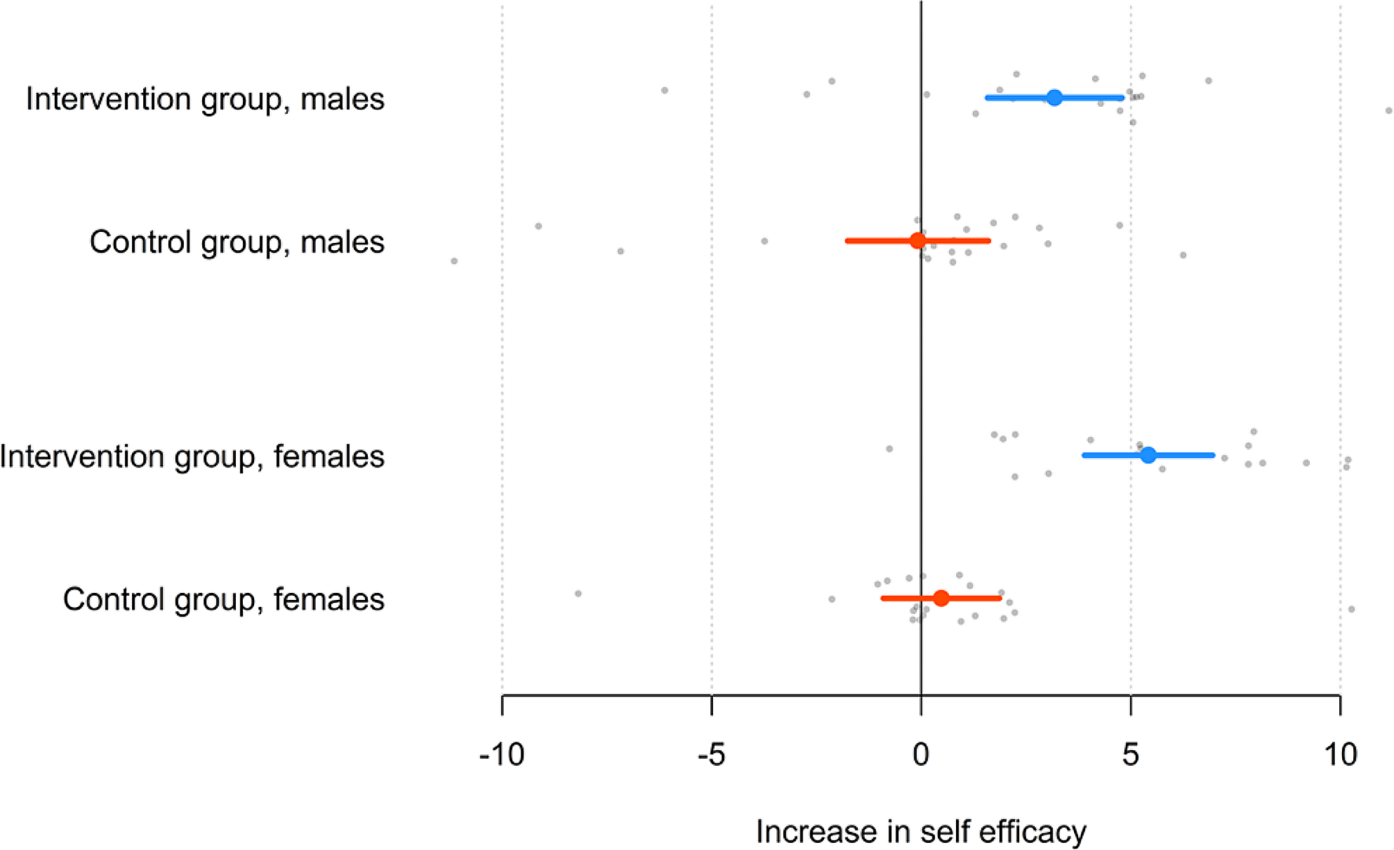
Increase in self-efficacy for the intervention and control groups for male and female students Note: The circle is the mean, and the line is the 95% confidence interval OR quartiles, and grey dots are individual students

Knowledge increased by an average of 1.52 points for all female students (*t*_39_ = 2.55, *p* =.015) and by an average of 0.63 points for all male students (*t*_45_ = 1.54, *p* =.13); the difference was not significant (*t*_84_ = 1.26, *p* =.21). For the female students, the mean increase in knowledge was 2.42 in the intervention group (*t*_18_ = 2.65, *p* =.016) and 0.71 in the control group (*t*_20_ = 0.94, *p* =.36); again, the difference was not significant (*t*_38_ = 1.44, *p* =.16). For the male students, the mean increase in knowledge was 0.64 in the intervention group (*t*_21_ = 1.88, *p* =.07) and 0.63 in the control group (*t*_23_ = 0.86, *p* =.40); again, the difference was not significant (*t*_44_ = 0.014, *p* =.99), see Figs. 3 and 4.

Self-efficacy increased by an average of 2.82 points for all of the female students (*t*_39_ = 4.52, *p* <.05) and by an average of 1.48 points for all male students (*t*_45_ = 2.44, *p* =.019); the difference was not significant (*t*_84_ = 1.54, *p* =.13). For the female students, the mean increase in self-efficacy was 5.42 in the intervention group (*t*_18_ = 7.46, *p* <.05) and 0.48 in the control group (*t*_20_ = 0.72, *p* =.48); the difference was significant (*t*_38_ = 5.03, *p* <.05). For the male students, the mean increase in self-efficacy was 3.18 in the intervention group (*t*_21_ = 4.12, *p* <.05) and − 0.083 in the control group (*t*_23_ = 0.10, *p* =.92); again, the difference was significant (*t*_44_ = 2.90, *p* =.0058). This indicates that the two groups (males and females) significantly differed in their self-efficacy scores after the flipped learning intervention, see Fig. 4.

To test whether the effect of the intervention on the increase in knowledge and self-efficacy varied by gender, a linear model with an interactive term is used. Table 3 shows the estimated coefficients for this model, with standard errors in parentheses. The *F* test for this model found no significant evidence that any of these variables had affected the change in knowledge (*F*_3,82_=1.45, *p* =.24). For self-efficacy, the *F* test for this model was significant (*F*_3,82_=11.02, *p* <.05), which indicates that one or more of the variables in this model had significantly affected the change in self-efficacy. Table 3 shows that the coefficient for the intervention was significant (*t*_82_ = 4.46, *p* <.05). This coefficient represents the effect of the intervention among the female students, that is, the difference between the self-efficacy increase of the female students in the flipped group. This implies that the female learners in the flipped group had an improvement in their perceived self-efficacy after the intervention compared with their female counterparts in the control group. However, the interaction coefficient was the coefficient of primary interest in this model because it was the primary reason for estimating this model. The estimated interaction coefficient was − 1.68, which means that the intervention had increased self-efficacy by 1.68 points more among the female students than it had among the male students (4.94 increase among the females and 3.26 among the males). Even so, this interaction coefficient was not significant possibly due to the small sample sizes (*t*_82_ = 1.11, *p* =.27). This means there is no significant evidence that the intervention had a greater effect on the female students than it did on the male students.

**Table 3:** Coefficients of the linear models, estimated with Ordinary Least Squares

	Dependent variables	
	*Knowledge increase*	*Self-efficacy increase*
Intervention	1.707 (1.033)	4.945*** (1.108)
Male	-0.089 (0.975)	-0.560 (1.046)
Intervention * Male	-1.695 (1.431)	-1.680 (1.515)
Constant	0.714 (0.712)	0.476 (0.764)
Observations R^2^ Adjusted R^2^ Residual Std. Error (df = 82)	86 0.050 0.015 3.264	86 0.287 0.261 3.501
F Statistics (df = 3;82)	1.445	11.017***

Note: *p < 0.1; **p < 0.05; ***p < 0.01

### Students’ perceptions

Students’ perceptions about their experience of learning haematology with the flipped learning instruction were mostly favourable. All the ten survey items were agreed or strongly agreed with by at least 69% of the 41 students. The two items with the highest percentages of agreement from the students were “viewing the lecture before scheduled class prepared me for the class activity” (92%, *M* = 4.59, SD = 0.74) and “viewing the pre-recorded lecture was essential to successfully participating in the class activity” (90%, *M* = 4.48, SD = 0.86). Most students (86%) also agreed about their confidence to address haematology topics in the final test (*M* = 4.30, SD = 0.78). Similarly, 84% of the students agreed they need more interaction between students and faculty members in class (*M* = 4.21, SD = 0.70). Additionally, although 70% of students indicated that they viewed the video lecture before class (*M* = 3.66, SD = 1.07), 30% of them did not. Regarding the two statements on the instructor role in the flipped learning environment, the students agreed that the instructor made meaningful connections between the topics in the pre-recorded lecture and the class activity (*M* = 4.24, SD = 0.68) and required student participation in the in-class activity (*M* = 4.14, SD = 0.83). However, nearly quarter of the students (25%) indicated their neutral view of the statement “I enjoyed being able to view the lecture prior to schedule class as opposed to live class lecture” (*M* = 3.24, SD = 0.99), suggesting that some students might still be unsure about their preference of video lectures at home over live face-to-face lectures. Most students (78%) agreed that flipped learning instruction is far more dissimilar to the traditional instruction (*M* = 3.90, SD = 0.85). and 87% expressed a desire for more instructors to use the flipped learning model (*M* = 4.34, SD = 0.78). For full descriptive statistics, see Appendix F.

## Discussion

This study aimed to evaluate the impacts of a flipped classroom model on medical students’ academic performance and self-efficacy in a haematology course, and explore differences based on gender. A key finding was that the flipped learning group demonstrated significantly greater increases in self-efficacy compared to the traditional lecture group. Although academic test scores also improved more for the flipped group, the difference was not statistically significant.

The significant boost in self-efficacy is an important outcome, considering that self-efficacy beliefs can influence students’ motivation, effort, persistence, and academic achievement [12]. By providing more opportunities for active learning and peer/instructor interactions during class time, flipped instruction may empower students and strengthen their confidence to apply knowledge and skills. This aligns with previous studies showing self-efficacy improvements with flipped learning [26, 38]. Developing self-efficacious health professionals is critical as it can impact the quality of patient care they later provide.

Although academic gains were not significant between groups, the flipped class did show a small improvement over traditional teaching. The lack of significance could be due to the brief 10-week intervention and students’ adjustment to a new instructional approach. As students become more accustomed to taking responsibility for pre-class learning, academic effects may strengthen over time. Longer interventions and randomised trials on flipped instruction in hematology courses would lend more definitive evidence.

An additional key finding was that female students demonstrated greater improvements than male students on both outcomes, although differences were also non-significant. This trend of larger effects for females matches some prior studies on flipped classrooms [13, 54]. Potential explanations include gender differences in learning styles, study behaviors, or interactions within a gender-segregated environment. More research is needed on how gender influences engagement and success in flipped medical classrooms.

Overall, this study provides initial evidence that flipped instruction in haematology can positively impact an important affective outcome– health professions students’ self-efficacy beliefs. Although academic effects were less clear, students reacted positively to the interactive learning format. Flipping the classroom allows instructors to better meet diverse student needs and foster deeper engagement during precious in-class time. As the first study on a flipped haematology course for medical undergraduates in Saudi Arabia, it makes a meaningful contribution to understanding the student experience in this context. Findings can inform ongoing improvements to medical curricula in the country and beyond.

### Limitations

Several limitations might challenge the interpretation of the outcomes of the study. The limited sample size and short length of the investigation could potentially have affected the overall reliability and validity of the findings. In addition, this research was carried in the very specific context of Saudi Arabia and in the specific medical course of haematology, which could affect the generalisation of the results. To ensure the validity of the findings, future studies should incorporate randomisation techniques, have larger sample sizes and use more accurate tracking methodologies, such as qualitative, mixed and longitudinal approaches. These measures are necessary to elucidate the long-term impact of flipped learning on the process of knowledge acquisition. Only after conducting such studies can definitive conclusions be drawn.

As this study utilized a non-equivalent group pretest-posttest design without random assignment, changes from pre to post intervention could be influenced by confounding variables unrelated to the flipped classroom treatment. Maturation over the 10-week study period is a plausible alternative explanation if students’ self-efficacy improved simply due to their progression through the medical program. History effects could also play a role if other events occurred externally that boosted students’ confidence. And if the instructor had high expectations or was enthusiastic about flipped learning, his interpersonal manner with that group could have impacted attitudes.

To strengthen the evidence that observed gains were a direct result of the flipped classroom format, future studies should incorporate random assignment of participants to conditions. Using an equivalent control group from a comparable course or historical data would also control threats to validity. The inclusion of a qualitative component exploring students’ perceptions in depth could provide explanatory insights into mechanisms behind quantitative changes.

## Conclusions

The aim of this study was to investigate whether flipped learning impacts medical students’ academic achievement and self-efficacy in a haematology course, and whether effects differ by gender.

In terms of achievement outcomes, results showed no significant difference in gain scores between flipped classroom and traditional lecture groups. However, both conditions demonstrated small pre-post increases in haematology test performance. This indicates that while flipping the classroom did not diminish learning, it also did not confer clear academic advantages over customary instruction in this context. More research is needed into optimal configurations of pre-class and in-class activities to potentiate knowledge gains.

Notably, the flipped classroom students did report substantial improvements in self-efficacy over the control group. Quantitatively and qualitatively, they expressed greater confidence in applying haematological concepts and skills. This highlights an affective benefit of active learning models that warrant further investigation. Developing health professionals’ self-belief can support competence and motivation for lifelong practice.

Exploratory analyses also revealed a non-significant tendency for stronger learning and self-efficacy gains among female students, aligning with previous literature on gender differences in flipped classrooms. Due to sample size limitations, more research into the interactions between learner gender, engagement, and performance in flipped medical courses is justified.

In conclusion, introducing a flipped classroom model to replace traditional lectures does not necessarily guarantee improved academic success based on the results of this initial haematology experiment. However, benefits to supplemental outcomes like self-efficacy and female engagement intimated promise. Health professions educators could consider “flipping” select courses or units to provide variety in instructional modes and leverage active learning opportunities. But simply exporting pre-class content without intention toward purposeful in-person activities risks losing existing benefits of face-to-face education. Overall, there is still much to learn about best principles and practices for flipped learning in medical contexts.

### Electronic supplementary material

Below is the link to the electronic supplementary material.

Supplementary Material 1

## Data Availability

No datasets were generated or analysed during the current study.
