# Supplementary material for: Biomedical students’ self-efficacy and academic performance by gender in a flipped learning haematology course
Source: BMC Med Educ. 2024 Apr 24;24:443. doi: 10.1186/s12909-024-05421-2 (PMC11040756; doi:10.1186/s12909-024-05421-2)
Supplement: Supplementary file 1 — Supplementary Material 1 [file 12909_2024_5421_MOESM1_ESM.docx]

**Appendices**

Appendix A: Schedule of the Study and Haematology Content Covered

| *Week* | *Topics* |
| --- | --- |
| Week 1 | Pre-test of haematology  Pre-self-efficacy questionnaire  Training sessions for students in the flipped group |
| Week 2 | Components of blood cells |
| Week 3 | Components of blood cells |
| Week 4 | Identifying all organs involved in blood cell production |
| Week 5 | Identifying all organs involved in blood cell production |
| Week 6 | Integrating various blood cell types with their functions |
| Week 7 | Integrating various blood cell types with their functions |
| Week 8 | Laboratory procedures associated with blood evaluation |
| Week 9 | Laboratory procedures associated with blood evaluation |
| Week 10 | Post haematology test  Post self-efficacy survey  Students’ perceptions of the intervention |

Appendix B: Overview of the design. First Principles of Instruction (from Merrill, 2002, pp. 43-57)

| ***Instructional principles*** | ***Description*** | ***Implementation in the present study*** |
| --- | --- | --- |
| *Learning is promoted when new knowledge is demonstrated to the learner*  **(*Demonstration*)** | Learning is promoted when the teacher demonstrates the appropriate procedures to solve the problems | The instructor demonstrated the key knowledge of the topic through video lectures which the students had to watch before coming to the lecture  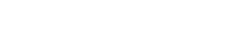 |
| Learning is promoted when existing knowledge is activated as a foundation for new knowledge  **(*Activation*)** | Learning is promoted when learners are directed to recall, relate, describe or apply knowledge from relevant or past experience which can be used as a foundation for new knowledge | Learning was guided by computerized graded feedback on the students’ response to online short quizzes following each video lecture. Additionally, at the start of each in-class lecture, the instructor reviewed the information materials as a foundation for the students learning new knowledge |
| *Learning is promoted when new knowledge is applied by the learner*  **(*Application*)** | Students have to use their new knowledge or skills to solve new problems. Additionally, the instructor should design and arrange problem-solving exercises as sequences of various challenges. | The students applied what they had learned from the video lectures to solve various problems. The instructor was available to provide them with individualised feedback.  During the lecture time, the students engaged in a variety of medical scenarios and practices |
| *Learning is promoted when new knowledge is integrated into the learner’s world*  **(*Integration*)** | Students should reflect on, discuss, defend and apply their new knowledge or skills | The students had time during the face-to-face lecture for questions and responses on the prior e-learning topics, and practised making diagnoses based on what they had learnt from the e-learning contents and their understanding from the continuing clinical practicum  The students shared and discussed their approaches to solving medical scenarios and case studies in the practical sessions. In addition, they were prompted to explain their medical reasoning to their instructor and peers. |

**Appendix C. Haematology achievement test**

Choose the correct answer for each of the following questions.

Note: please choose only **one** answer for each item.

1. Which of the following is NOT a likely aetiology for sideroblastic anaemia?
2. Isoniazid toxicity
3. Lead poisoning
4. Alcohol abuse
5. **Iron deficiency**
6. Myelodysplasia
7. Common causes of iron overload (haemosiderosis) include:
8. Increased iron absorption e.g. hereditary haemochromatosis
9. Increased iron intake
10. Repeated red cell transfusions
11. **All of the above**
12. None of the above
13. Which statement regarding iron overload (haemosiderosis) is correct:
14. There is no control mechanism for eliminating excess iron.
15. Iron overload can occur in disorders associated with excessive absorption.
16. Iron overload can occur in disorders associated with chronic blood transfusion
17. Excessive iron deposition in tissues can cause damage to organs.
18. **All of the above**
19. Which of the following conditions is most likely with significant deficiency of vitamin B12 or folate?
20. Decreased DNA synthesis
21. Macrocytosis
22. Megaloblastic anaemia
23. Decreased osteoblast activity
24. **All of the above**
25. Which of the following is NOT a feature of megaloblastic anaemia?
26. **Microcytic RBC**
27. Mean corpuscular volume > 100
28. Leukopenia
29. Thrombocytopenia
30. Presence of hypersegmented neutrophiles
31. Deficiency of which of the following could result in megaloblastic anaemia:
32. **Vitamin B12 deficiency / Folate deficiency**
33. Vitamin C deficiency
34. Vitamin D deficiency
35. Vitamin K deficiency
36. Vitamin B complex
37. Which of the following is true about orotic aciduria?
38. **Congenital enzyme deficiencies associated with megaloblastic anaemia**
39. Acquired enzyme deficiencies
40. Associated with sickle cell anaemia
41. Associated with thalassaemia
42. All of the above
43. Common causes of megaloblastic anaemia include:
44. Vitamin B12 deficiency / Folate deficiency
45. Abnormalities of vitamin B12 or folate metabolism
46. Congenital enzyme deficiencies
47. Acquired enzyme deficiencies
48. **All of the above**
49. The following are examples of haematological findings in megaloblastic anaemia:
50. Macrocytic anaemia
51. Thrombocytopenia
52. Leukopenia
53. Neutrophil nuclei hypersegmentation
54. **All of the above**
55. Which one of the following conditions would be included in the differential diagnosis of an anaemic adult patient with an MCV of 125 fL
56. Aplastic anaemia
57. Sickle cell anaemia
58. Iron deficiency
59. Sideroblastic anaemia
60. **Vitamin B12 deficiency**
61. The most common causes of folate deficiency are:
62. Nutritional
63. Malabsorption
64. Excess utilization
65. Excess urinary folate loss
66. **All of the above**
67. The macrocytosis associated with megaloblastic anaemia results from:
68. **Reduced number of cell divisions**
69. Increased number of cell divisions
70. Compromised RNA synthesis
71. G6PD deficiency
72. All of the above
73. Tests for the cause of folate deficiency include:
74. Diet history
75. Tests for intestinal malabsorption
76. Anti-transglutaminase and endomysial antibodies
77. Duodenal biopsy underlying disease
78. **All of the above**
79. Which one of the following is characteristic of pernicious anaemia (PA)?
80. PA caused by autoimmune attack on the gastric mucosa
81. More females than males are affected
82. A peak occurrence at 60 years
83. Helicobacter pylori infection can initiate an autoimmune gastritis
84. **All of the above**
85. Which of the following is the standard method to diagnose pernicious anaemia once cobalamin deficiency is confirmed?
86. **Schilling test**
87. CBC
88. Prothrombin time (PT)
89. HBA1C
90. Serum iron level
91. Regarding haemolytic anaemia, which of the following is correct:
92. Haemolytic anaemia results from an increase in the rate of RBC destruction
93. Shortening the RBC lifespan to less than 120
94. High reticulocyte count
95. Normocytic normochromic
96. **All of the above**
97. Which one of the following provides an assessment of the bone marrow’s response to anaemia?
98. Haemoglobin
99. Haematocrit
100. **Reticulocyte count**
101. Red cell distribution width (RDW)
102. Erythrocyte sedimentation rate (ESR)
103. Which of the following is correct with the classification of haemolytic anaemia?
104. Hereditary anaemia and acquired anaemia
105. Intravascular and extravascular
106. Intrinsic defect and extrinsic defect
107. **All of the above**
108. None of the above
109. Autoimmune haemolytic anaemias are associated with:
110. **Warm and cold antibodies**
111. Drugs
112. Alcohol
113. Infection
114. All of the above
115. The primary underlying mechanism in the pathogenesis of ACD is:
116. Decreased release of iron from macrophages to plasma
117. Reduced red cell lifespan
118. Inadequate erythropoietin response to anaemia caused by the effects of cytokines
119. **All of the above**
120. None of the above
121. Laboratory findings in extravascular haemolytic anaemia include:
122. Increased of serum bilirubin
123. Increased of urine urobilinogen and/or faecal stercobilinogen
124. Serum haptoglobin absent
125. Reticulocytosis, spherocytes, elliptocytes
126. **All of the above**
127. Defects in RBC membrane in hereditary spherocytosis include:
128. Spectrin
129. Ankyrin
130. Band 3 protein
131. Protein band 4.2 abnormalities
132. **All of the above**
133. Which abnormal RBC morphology is typically observed on the peripheral blood film in haemolysis mediated by IgM antibodies?
134. Neutrophil
135. Microcytosis
136. **RBC agglutination**
137. Lymphocyte
138. None of the above
139. The destruction of red blood cells is called:
140. **Haemolysis**
141. Glycolysis
142. Oval cytosis
143. Homeostasis
144. Haemostasis
145. What is thalassaemia?
146. An autoimmune disorder
147. **A genetic blood disorder**
148. A viral infection
149. A type of cancer
150. All of the above
151. Which type of thalassaemia involves a mutation in the alpha-globin gene?
152. **Alpha thalassaemia**
153. Beta thalassaemia
154. Delta thalassaemia
155. Gamma thalassaemia
156. Which type of thalassaemia involves a mutation in the beta-globin gene?
157. Alpha thalassaemia
158. **Beta thalassaemia**
159. Delta thalassaemia
160. Gamma thalassaemia
161. All of the above
162. In alpha thalassaemia, the severity of the disease depends on:
163. **The number of mutations in the alpha-globin gene**
164. The number of mutations in the beta-globin gene
165. The presence of an additional delta-globin gene
166. The level of iron in the blood
167. None of the above
168. Haemoglobin lepore is characterized by:
169. Reduced production of alpha-globin chains
170. Reduced production of beta-globin chains
171. Abnormal fusion of the alpha-globin and delta-globin genes
172. Abnormal fusion of the beta-globin and gamma-globin genes
173. **All of the above**
174. Bart’s haemoglobin is a result of:
175. **Complete loss of four alpha globin genes**
176. Complete loss of beta-globin genes
177. Loss of two alpha globin genes
178. Loss of one beta globin gene
179. None of the above

**Appendix D.** **Haematology pre-test**

Choose the correct answer for each of the following questions?

Note: please choose only **one** answer for each item.

1. Where is most of the body's iron contained?
2. Hemosiderin in the reticuloendothelial system
3. **Haemoglobin in RBC**
4. Transferrin in plasma
5. Myoglobin in muscle
6. Ferritin in the reticuloendothelial system
7. Which amino acid is involved in the synthesis of heme?
8. Leucine
9. Methionine
10. **Glycine**
11. Arginine
12. Lysine
13. The process of formation and development of blood cells is termed:
14. **Haematopoiesis**
15. Haematemesis
16. Haematocytometry
17. Haematorrhea
18. Erythropoiesis
19. During 2-7 months of foetal development, the site of blood cell production is the:
20. Bone marrow
21. Yolk sac
22. Lymph nodes
23. **Liver and spleen**
24. Kidney
25. In adults, haematopoiesis of red blood cells and platelets occurs primarily in:
26. **Bone marrow**
27. Liver
28. Spleen
29. Yolk sac
30. All of the above
31. Physiologically programmed cell death is known as:
32. Angiogenesis
33. **Apoptosis**
34. Aneurysm
35. Apohematics
36. Leukopoiesis
37. A haemoglobin molecule is composed of:
38. One heme molecule and four globin chains
39. Ferrous iron, protoporphyrin IX and a globin chain
40. **Four heme molecules and four globin chains**
41. Protoporphyrin IX and four globin chains
42. One heme molecule and one globin chain
43. The predominant haemoglobin found in a healthy adult is:
44. Gower-1
45. A2
46. **A**
47. F
48. A2
49. Iron is transported in plasma via:
50. Haaemosiderin
51. Ferritin
52. **Transferrin**
53. Haemoglobin
54. Albumin
55. Concerning haemoglobin, which one of the following is correct?
56. Adult haemoglobin contains four identical globin chains
57. **Each haemoglobin can carry four molecules of oxygen**
58. Oxygen binding follows a hyperbolic curve
59. 2,3-diphosphoglycerate levels rise in hypoxia to allow increased oxygen uptake by haemoglobin
60. The Bohr effect (a shift of the oxygen dissociation curve to the right) is due to decreased H^+^ concentration.
61. Regarding erythrocytes, which one of the following is correct?
62. They have a bi-lobed nucleus
63. They are derived from lymphoid progenitor cells
64. **RBC transports CO^2^**
65. They have an average lifespan of 12 days
66. They have a spherical shape
67. The earliest morphologically recognizable erythrocyte precursor is:
68. **Proerythroblast**
69. Hamatopoietic stem cell (HSC)
70. Neutrophil
71. Platelets
72. None of the above
73. Hypoxia stimulates RBC production by:
74. Inducing more pluripotent stem cells into the erythroid lineage
75. **Stimulating EPO production by the kidney**
76. Increasing the number of RBC mitoses
77. Stimulating the production of fibronectin by macrophages of the bone marrow
78. All of the above
79. What is erythropoiesis?
80. Formation of platelets
81. **Formation of red blood cells**
82. Formation of white blood cells
83. Formation of plasma proteins
84. All of the above
85. Where does erythropoiesis occur in adults?
86. Liver
87. Lungs
88. **Bone marrow**
89. Spleen
90. All of the above
91. What hormone stimulates erythropoiesis?
92. Insulin
93. Thyroxine
94. **Erythropoietin**
95. Cortisol
96. Testosterone
97. Which of the following nutrients is essential for erythropoiesis?
98. Vitamin C
99. Vitamin D
100. **Iron**
101. Calcium
102. Zinc
103. Which cell type is the precursor for erythrocytes?
104. Lymphocyte
105. Monocyte
106. Megakaryocyte
107. **Proerythroblast**
108. Neutrophil
109. What is the lifespan of a mature erythrocyte?
110. 60 days
111. **120 days**
112. 180 days
113. 240 days
114. 280 days
115. What happens to erythrocytes at the end of their lifespan?
116. **They are destroyed by macrophages**
117. They are transformed into platelets
118. They are transformed into white blood cells
119. They are excreted through urine
120. They are transformed into macrophages
121. What is the function of erythrocytes?
122. **To transport oxygen and carbon dioxide**
123. To fight infections
124. To clot blood
125. To produce antibodies
126. To stop bleeding
127. What is the condition called when there is a deficiency of erythrocytes in the body?
128. **Anaemia**
129. Leukaemia
130. Thrombocytopenia
131. Haemophilia
132. All of the above
133. What is the name of the hormone produced by the kidneys which stimulates erythropoiesis?
134. Testosterone
135. Oestrogen
136. Thyroid hormone
137. **Erythropoietin**
138. Progesterone
139. What is the protein that makes up the majority of red blood cells and is responsible for carrying oxygen?
140. **Haemoglobin**
141. Myoglobin
142. Albumin
143. Fibrinogen
144. Thrombin
145. What is the name of the metal ion found in the centre of the heme group in haemoglobin?
146. **Iron**
147. Copper
148. Zinc
149. Calcium
150. None of the above
151. What is the process called by which white blood cells are produced in the body?
152. Haematopoiesis
153. Erythropoiesis
154. **Leukopoiesis**
155. Thrombopoiesis
156. Apoptosis
157. What is the process called by which blood cells are produced in the body?
158. **Haematopoiesis**
159. Erythropoiesis
160. Leukopoiesis
161. Thrombopoiesis
162. None of the above
163. What is the name of the stem cell that gives rise to all types of blood cell?
164. Myeloid stem cell
165. Lymphoid stem cell
166. Erythroid stem cell
167. Megakaryocyte
168. **Pluripotent stem cell**
169. What is the primary function of apoptosis?
170. To remove damaged or unnecessary cells from the body
171. To create new cells in the body
172. **To maintain cellular homeostasis**
173. To produce energy for the cell
174. All of the above
175. What is the primary function of haematopoietic stem cells (HSCs)?
176. To produce red blood cells
177. To produce white blood cells
178. To produce platelets
179. **To produce all types of blood cell**
180. None of the above

**Appendix E. Descriptive statistics of the comparison of mean scores for responses to statements reflecting items of students Self-Efficacy Questionnaire at the end of the study (n=86)**

This survey contains 23 statements about your confidence in doing things related to haematology. For each question, think about how confident you would be in carrying out a given task.

| **Items** | **Flipped group**  **Mean (SD)**  **n=41** | **Traditional group**  **Mean (SD)**  **n=46** |
| --- | --- | --- |
| 1. How confident are you that after reading an article about a haematology experiment, you could write a summary of its main points? | 4.10(1.08) | 3.87(1.10) |
| 2. How confident are you that you could critique a haematology laboratory report written by another student? | 4.27(0.80) | 3.96(.95) |
| 3. How confident are you that you could write an introduction to a haematology lab report? | 3.93(1.16) | 3.49(1.16) |
| 4. How confident are you that after reading an article about a haematology experiment, you could explain its main ideas to another person? | 3.15(1.12) | 3.58(1.36) |
| 5. How confident are you that you could read the procedures for an experiment in a haematology lab and feel sure about conducting the experiment on your own? | 4.01(.956) | 3.69(0.85) |
| 6. How confident are you that you could write the methods section of a haematology lab report (i.e., describe the experimental procedures)? | 3.95(1.17) | 2.71(0.84) |
| 7. How confident are you that after watching a television documentary dealing with some aspect of haematology, you could write a summary of its main points? | 3.32(1.07) | 3.31(1.24) |
| 8. How confident are you that you will be successful in this haematology course? | 4.05(1.06) | 3.56(1.22) |
| 9. How confident are you that you could write up results to a lab report? | 3.80(1.04) | 3.64(1.19) |
| 10. How confident are you that after watching a television documentary dealing with some aspect of haematology, you could explain its main ideas to another person? | 3.88(1.23) | 3.00(1.37) |
| 11. How confident are you that you will be successful in another haematology course? | 3.02(1.22) | 3.36(1.21) |
| 12. How confident are you that you could write the conclusion to a lab report? | 3.85(1.16) | 3.84(0.93) |
| 14. How confident are you that after listening to a public lecture regarding some haematology topic, you could write a summary of its main points? | 3.73(1.13) | 3.31(1.14) |
| 15. How confident are you that you could analyse a set of data (i.e., look at the relationships between variables)? | 3.95(0.91) | 3.38(1.32) |
| 16. How confident are you that after listening to a public lecture regarding some haematology topic, you could explain its main ideas to another person? | 3.88(1.25) | 3.60(1.05) |
| 17. How confident are you that you would be successful in a clinical haematology rotation course? | 4.17(0.93) | 2.76(0.91) |
| 18. How confident are you that you could tutor another student on how to write a lab report in haematology? | 3.24(1.10) | 3.16(1.22) |
| 19. How confident are you that you could critique an experiment described in a haematology textbook (i.e., list the strengths and weaknesses)? | 3.83(1.10) | 3.31(1.16) |
| 20. How confident are you that you could tutor another student for this haematology course? | 3.61(1.08) | 3.42(1.29) |
| 21. How confident are you that you could ask a meaningful question that could be answered experimentally? | 3.56(1.06) | 2.53(1.25) |
| 22. How confident are you that you could recall skills to collect and process specimens for the investigation of a haematological disease? | 4.00(1.19) | 3.27(1.16) |
| 23. How confident are you that you could determine the principle, methodology and application of different haematological techniques? | 4.17(0.19) | 3.21(1.11) |

**Appendix F. Descriptive statistics of students’ perception of the flipped learning group (n=41).**

| **Statements** | **Descriptive** | |
| --- | --- | --- |
|  | ***M*** | ***SD*** |
| 1. Viewing the lecture before the scheduled class prepared me for the class activity. | 4.59 | 0.74 |
| 1. I did not view the lecture before the class although I was supposed to. | 3.66 | 1.07 |
| 1. Viewing the pre-recorded lecture was essential for successfully participating in the class activity. | 4.48 | 0.86 |
| 1. The instructor made meaningful connections between the topics in the pre-recorded lecture and the class activity. | 4.24 | 0.68 |
| 1. The flipped classroom model was similar to other classes in the Bernard J. Dunn School of Pharmacy. | 3.90 | 0.85 |
| 1. I enjoyed being able to view the lecture prior to the scheduled class as opposed to a live class lecture. | 3.24 | 0.99 |
| 1. The instructor required student participation in the in-class activity. | 4.14 | 0.83 |
| 1. I am confident about my ability to address these topics in the final examination. | 4.30 | 0.78 |
| 1. I want more interaction between students and instructors in class. | 4.21 | 0.70 |
| 1. I wish more instructors used the flipped classroom model. | 4.43 | 0.78 |
